# Prenatal and Postnatal Anxiety and Depression in Mothers during the COVID-19 Pandemic

**DOI:** 10.3390/jcm10143193

**Published:** 2021-07-20

**Authors:** Joanna Baran, Justyna Leszczak, Rafał Baran, Anna Biesiadecka, Aneta Weres, Ewelina Czenczek-Lewandowska, Katarzyna Kalandyk-Osinko

**Affiliations:** 1 Institute of Health Sciences, Medical College, University of Rzeszów, Al. mjr. W. Kopisto 2a, 35-310 Rzeszów, Poland; leszczakjustyna.ur@gmail.com (J.L.); anetaweres.ur@gmail.com (A.W.); ewczen@wp.pl (E.C.-L.); 2 Natural and Medical Center for Innovative Research, ul. Litawora 2, 35-310 Rzeszów, Poland; 3 SOLUTION-Statistical Analysis, ul. Stojałowskiego 4/73, 35-120 Rzeszów, Poland; rafal.barann@gmail.com; 4 PELVIMED mgr Anna Biesiadecka, Aleja Niepodległości 4/32, 39-300 Mielec, Poland; pelvimedrzeszow@gmail.com; 5 Institute of Medical Science, Medical College, University of Rzeszów, Al. mjr. W. Kopisto 2a, 35-310 Rzeszów, Poland; kalandyk@op.pl; 6 Department of Gynecology and Obstetrics of the Frederic Chopin Clinical Provincial Hospital No. 1 in Rzeszów, ul. Szopena 2, 35-055 Rzeszów, Poland; 7 Fortitudo Medical Center, ul. Jana Pawła II 94, 35-317 Rzeszów, Poland

**Keywords:** anxiety, COVID-19, loneliness, postpartum depression

## Abstract

The aim of this study was to assess the changes in the occurrence of prenatal and postnatal anxiety and depression symptoms, and to assess what factors significantly affect the appearance of symptoms of depression and anxiety in young mothers. The study group consisted of 130 women after childbirth. Due to the ongoing restrictions caused by the COVID-19 pandemic, the survey was prepared online. The questionnaire was fully anonymous, and it contained the authors’ own questions and two standardized questionnaires: the Edinburgh Postnatal Depression Scale (EPDS) and Generalized Anxiety Disorders GAD-7. The conducted analysis clearly indicated that the level of postpartum depression, in as many as 52 of the mothers, had increased significantly compared to the time before delivery, when symptoms of depression were shown by 22 women (*p* = 0.009). However, there was no statistically significant change between prenatal and postnatal anxiety. There are many factors associated with postnatal depression. The strongest predictors turned out to be average socioeconomic status, history of anxiety disorders, past neurosis or depression, lack of or inadequate level of assistance from healthcare professionals, as well as lactation problems and postpartum pain.

## 1. Introduction

Postpartum depression (PPD) is a condition that affects many mothers. It can appear even before a birth and up to one year after a birth [1]. The enormity of changes taking place in the woman’s body after childbirth [2], hormonal changes [3], the need to get used to the new situation, caring for the child, fatigue, and lack of sleep [4] are only some of the factors contributing to the emergence of depression and anxiety. According to Fiala et al., the main risk factors for the development of PPD identified as significant at all three time points (before delivery, 6 weeks after delivery, and 6 months after delivery) were a personal history of previous depressive episodes and a mother who had experienced significant psychosocial stressors [5].

Depression negatively affects not only the health of the mother but also that of the baby [6]. Infants and pre-schoolers of depressed mothers are at risk of poor self-control, problems with internalization and externalization, and difficulties in cognitive functioning and social contact with parents and peers [7]. Babies of depressed mothers may show developmental abnormalities at 3 years of age [8] or cognitive defects at 4 years [9]. School-age children and adolescents of parents suffering from depression are at risk of impaired adaptive functioning and psychopathology, including behavioral disorders, affective disorders, and anxiety disorders. They are also at risk of ADHD and learning disabilities [10]. In addition, the mother’s depression may negatively affect her relationship with her child [11,12].

The outbreak of the COVID-19 pandemic has drastically changed lives around the world. The spreading novel virus, the lack of effective means of treatment, and the constantly emerging new symptoms and complications have led successive countries to introduce ever-increasing restrictions aimed at reducing the spread of the virus [13]. One such restriction is, for example, a ban on visiting hospitals, including family births and hospital visits after a birth [14,15].

At this difficult time, a new mother may be left alone, dependent only on the assistance of medical staff, who, having many new mothers and babies in their care, cannot always provide assistance to the extent that a young mother needs. Postpartum pain, fatigue, problems with feeding or caring for the baby additionally contribute to frustration and malaise. In addition, apart from physical assistance, the lack of contact with a close family member is detrimental [16,17].

In addition to symptoms of depression, generalized anxiety disorder (GAD) may also appear during pregnancy. Its incidence varies between 8.5% and 10.5% [18]. Anxiety, known as ἄγχος by the ancient Greeks, was described by the Danish philosopher Søren Kierkegaard in The Concept of Anxiety in 1844 [19] and later by Sigmund Freud, who identified the concept of generalized, persistent, and free-floating anxiety, subsequently included in classification systems as a generalized anxiety disorder in 1980.

Untreated GAD can lead to pregnancy complications such as low birth weight, preterm labor, high blood pressure, problems with the baby’s neurological development, and a lack of progress in labor [20]. After the baby is born, the mother may also find it difficult to cope with the newborn’s demands and develop a bond with her baby [21].

Most current studies have found an increased incidence of prenatal anxiety and depressive symptoms among pregnant women during the COVID-19 pandemic, and many have reported a fear of giving birth during this time [22,23,24].

The aim of this study was to measure the changes in the occurrence of prenatal and postnatal anxiety and depression symptoms and to assess what factors significantly affect the appearance of symptoms of depression and anxiety in young mothers.

## 2. Materials and Methods

### 2.1. Participants

The research was conducted in the period from July to September 2020. An invitation to participate in the study was sent to 350 women. A total of 328 confirmed their participation in the study, of which only 130 completed the entire questionnaire and were qualified as the study group. The remaining women started filling in the questionnaire but failed to complete it, therefore, their results were not taken into account.

The average age of the respondents was 31 years old and their average bodyweight was almost 66 kg. The women gained an average weight of 14 kg (maximum 30 kg) during pregnancy. The babies were born between 33 and 43 weeks. The women rated their pain on a scale of 0 to 10, and the mean value was 6. Detailed data are presented in Table 1.

### 2.2. Methods

Due to the limitations on visits to doctors’ surgeries caused by the COVID-19 pandemic at that time, the survey was prepared online. The questionnaire was fully anonymous and completing it conveyed consent to participate in the study. It contained the authors’ own questions concerning the characteristics of the respondent (e.g., age, education, socioeconomic status, type of childbirth, GWG) and two standardized questionnaires: the Edinburgh Postnatal Depression Scale (EPDS) and Generalized Anxiety Disorders GAD-7.

The level of education was determined on the basis of the stages of schooling that the participant had completed during their education in Poland. The first stage lasts 8 years (from 7 to 15 years of age) and covers basic education (there was no respondent in this group, therefore, it was omitted from the analysis). The second stage is a 4-year secondary school (15 to 19 years of age) and may be general education (leading to a secondary education qualification) or job-oriented (leading to a vocational education qualification for a specific profession). The third stage consists of studies, e.g., at university, technical university, maritime academy, etc. (leading to a higher education qualification). Socio-economic status (SES) was subjectively reported by the respondents.

The EDPS questionnaire is the most widely used screening tool for depression in perinatal care [25,26]. It was developed by Cox et al. [27] in 1987 and consists of 10 questions relating to the previous 7 days. The answers are scored on a scale of 0 to 3. Additionally, in order to avoid answer option order bias, in some questions, the answers are arranged according to the score 0, 1, 2, 3, and the remaining 3, 2, 1, 0. In the present study, the cut-off points according to the latest research by Levis et al. [28] were used. The authors found that 11 or more points showed symptoms of postpartum depression.

The GAD-7 questionnaire is a useful tool with strong criteria validity for identifying probable cases of generalized anxiety disorder. The scale is also an excellent measure of severity, as evidenced by the fact that rising GAD-7 scores are strongly associated with multiple domains of functional impairment and disability. The cut-off points 5, 10, and 15 can be interpreted as representing moderate, moderately severe, and severe levels of anxiety in GAD-7. GAD-7 may be particularly useful in assessing the severity of symptoms and monitoring their changes over time [29].

On the GAD-7 scale, seven items were selected to assess the women’s feelings: being anxious or upset; the ability to avoid or control unease; worrying about various things; trouble relaxing; unease; being easily upset or irritated; feeling scared as if something terrible might happen. GAD-7 is also used for other anxiety disorders [30,31].

### 2.3. Statistical Analysis

The obtained data were statistically analyzed. Descriptive statistics (N, mean, minimum, maximum, Q_1_, Me, Q_3_, SD) were determined. The Chi test was used to assess the occurrence of particular levels of prenatal and postnatal depression and generalized anxiety in the mothers. In order to check the normality of the distribution, the Shapiro-Wilk test was carried out, which showed that the distribution of variables does not show features of normality. Therefore, non-parametric tests were used. On the other hand, to assess the relationship between the level of anxiety or depression depending on the selected factors, the *t*-test for dependent samples or Wilcoxon-test was used. The level of statistical significance was *p* < 0.05.

## 3. Results

**Figure 1:**
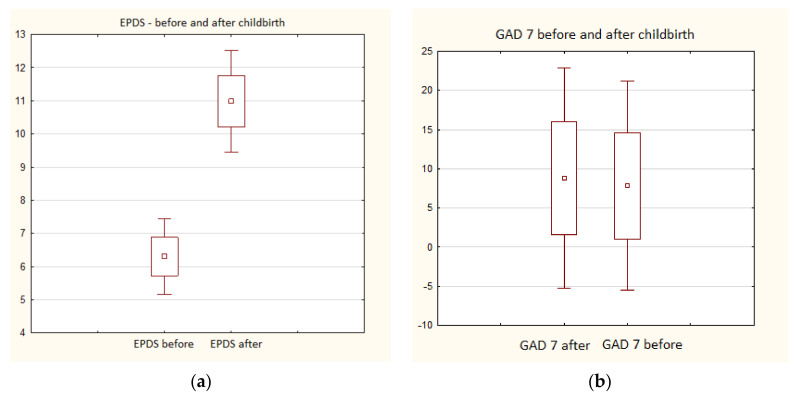
Level of prenatal and postnatal: depression (**a**) and anxiety (**b**) in the study group. ▫—mean; □—mean ± standard error; Ι—mean ± 1.96 standard error.

The conducted analysis clearly indicated that the level of postpartum depression among the mothers had increased significantly compared to the time before delivery (Figure 1a). Symptoms of prenatal depression were shown by 22 of the women, and postnatal depression in as many as 52 (*p* = 0.009) (Table 2).

However, there was no statistically significant change between prenatal and postnatal anxiety (Figure 1b). Although the number of women with moderate anxiety decreased and the number of women with severe anxiety increased, these results were not statistically significant (*p* = 0.330) (Table 3).

Figure S1a–j presents detailed results from individual items of the EPDS scale. The data compare pre-and post-natal responses. These results clearly indicate a deterioration of the women’s mental well-being after childbirth. This manifested itself, inter alia, in a reduction of the frequency of smiling and seeing the positive sides of life, a reduced tendency to look ahead with hope, blaming oneself for failure, feeling anxious and uneasy for no apparent reason, and an increase in the feeling of being overwhelmed in certain situations. It is also very significant that the women much more often felt lonely, sad, and cried (Figure S1a–j).

Figure S2a–g presents detailed results from individual items of the GAD-7 scale. The data compare pre-and post-natal responses. It can be stated that after delivery, the women assessed themselves as more nervous, unable to stop worrying, with difficulty in relaxing; however, the differences between the pre-delivery and postpartum examination were not statistically significant (Figure S2a–g).

In the final stage, an extensive analysis of the influence of various factors on the change of prenatal and postnatal anxiety and depression levels in the mothers was performed. In the case of anxiety, it was found that only those who had problems with lactation after childbirth and those with significant pain that made it difficult to care for their child showed a statistically significant increase in the level of anxiety (*p* = 0.004 and *p* = 0.037, respectively).

In the case of depression, there were many more factors. Symptoms of postpartum depression (score ≥ 11 points on the EPDS scale), which were not present before delivery, were reported in women with higher education (*p* < 0.001), manual workers (*p* = 0.021), from an urban environment (*p* < 0.001), with average socioeconomic status (*p* = 0.004), and with anxiety (*p* = 0.020), neurotic (*p* = 0.012), and depressive disorders (*p* = 0.019), giving birth during the COVID-19 pandemic (*p* < 0.001), cesarean section (*p* < 0.001), who were unable to count on visits from relatives in the hospital after childbirth (*p* < 0.001), or on assistance from staff (*p* < 0.001), or this assistance was given only occasionally (*p* = 0.003). Pain after childbirth that made it difficult to care for the child also played a significant role (*p* < 0.001) as well as problems with lactation after delivery (*p* < 0.001).

All of the results are presented in detail in Table 4.

## 4. Discussion

Postnatal depression is a mood disorder that occurs within a few months to a year after a birth. The causes of PPD can be physiological, situational, or multi-factorial [32].

The COVID-19 pandemic represented a significant risk factor for mental distress, especially anxiety, among women in the pregnancy or perinatal period. The psychological and social consequences may be equally upsetting. Women during quarantine have been physically isolated from family, friends, community, and schools [33].

Other concerning issues are the emotional, physical, and mental exhaustion and loss of medical staff, the shortage of essential hygiene equipment, the varieties of symptoms and secondary diseases caused by the infection, and the failure of many proposed treatments [34].

The study showed that there is a real difference in the women’s moods between pregnancy and the postpartum period. An analysis of the influence of selected factors that could potentially affect the occurrence of postpartum depression was undertaken. The factors were: socio-demographic (education of the respondents, place of residence, type of work, socioeconomic status), medical (previous history of depression, anxiety symptoms, neurosis, or bipolar disorder), and those directly related to childbirth (type of childbirth, occurrence of problems with lactation, postpartum pain). An important factor that was analyzed was childbirth during the prevailing COVID-19 pandemic and the associated limitations of visits to hospital wards and assistance of medical staff after delivery.

Each of the above-mentioned factors turned out to be significant in the appearance of postpartum depression symptoms in mothers.

Marital status, unwanted pregnancy, unexpected gender, infant disease, and low social support were independent predictors of postpartum depression [35]. Studies available in the literature indicate a high incidence of postpartum depression and post-natal symptoms in women who gave birth in a hospital at an epicenter of the COVID-19 pandemic. Psychological distress was mainly related to the level of pain experienced during childbirth or the perception of support from medical staff [36].

In the comments of the questions, many women complained that it was impossible to make up for the lack of a loved one after giving birth. The women reported that despite the great efforts of the medical staff, nothing could replace the presence of the partner, the father of the child. This especially made many of them feel powerless and helpless, cry, and feel that they were unable to cope with the baby alone. These women harbored hope and firmly believed that the situation would change after they returned home.

Literature data indicate that the most significant risk factors for developing PPD were a history of prior depression [37], stressful life events, childcare stress, and prenatal anxiety [32]. Our research confirmed that previous episodes of depression were conducive to the occurrence of PPD.

According to the path analysis model, postnatal depression is influenced by many factors, such as age, years of study, occupation, living conditions, and quality of life [38]. Feeding infants with formula or a mixture of breast milk and formula milk made mothers more susceptible to postpartum depression than those who only breastfed. In addition, if the infant’s weight gain was insufficient, mothers were more prone to concern about the health and development of their children compared to those whose infant was of normal weight [39].

In our study, one of the most important factors in the development of postpartum depression symptoms was Cesarean delivery. Other studies support this thesis [40,41]. It may be related to the greater limitations than after natural childbirth, pain at the site of the surgical wound, or delayed lactation.

In our study, lactation problems indicated a likelihood of symptoms of PPD, while other researchers’ opinions are divided [42]. Screening for depression during pregnancy can be a useful tool to identify women at risk of both shorter breastfeeding and postpartum depression. Experiencing breastfeeding problems may also expose women to symptoms of depression in the postpartum period [43], though Haga and colleagues showed that there was no evidence of a relationship between depressive symptoms and breastfeeding [44].

Antenatal state and anxiety as assessed by an interview is an important predictor of postpartum depression. Therefore, it should be routinely tested to develop specific preventive interventions [45].

Our study also showed that women with lactation problems or postpartum pain making it difficult to care for the baby showed symptoms of anxiety disorders. These disorders appeared before delivery and became significantly worse after delivery.

It is very common to experience increased unease during pregnancy and the postpartum period. Despite a decline in anxiety levels after childbirth, one woman in five is very anxious after childbirth. Anxiety and PPD overlap, but since one in four anxious women do not suffer from depression, screening for mental health problems after childbirth should include both depression and anxiety [46].

Depressive and anxiety symptoms in pregnant women showed several distinct groups of trajectories that are related to the stress of pregnancy. A small number of women experienced high levels of depressive symptoms throughout their pregnancy, and anxiety as assessed early in pregnancy continued without sudden changes. Perceived stress should be assessed and relieved in pregnant women because of its association with other mental health problems [47].

Early mental health screening is important to help detect women at risk of adverse pregnancy outcomes. Additional care should be provided to groups of pregnant women, and the underlying mechanisms of disorders in high-risk pregnant women must be investigated. The results of this study provide useful information for the design of interventions aimed at pregnant women who may need assistance. The research should be continued by extending the results to the prenatal and postnatal periods.

Despite the authors’ concerted efforts, certain methodological limitations could not be avoided. First, the study was based on an online survey, rather than a personal interview. The reason was the SARS-COV2 virus pandemic and thus the limited possibility of contact with patients. Second, the a high number of women who refused or failed to complete the questionnaire. This was due to, on the one hand, the researcher’s lack of control over the completion of the questionnaire, and on the other hand, many respondents reported that the questionnaire was too long (it contained questions about the period before the pandemic and during the pandemic, and took about 15 min to complete). Third, largely due to the above issues, was the small size of the group. In future research, these factors should be taken into account in the planning and modification of the study plan to avoid such weaknesses.

## 5. Conclusions

There are many factors associated with postnatal depression. The strongest predictors turned out to be average socioeconomic status, history of anxiety disorders, past neurosis or depression, lack of or inadequate level of assistance from healthcare professionals, as well as lactation problems and postpartum pain.

## Figures and Tables

**Table 1:** Characteristics of the study group.

Variables	N	x	Min.	Max.	Q_1_	Me	Q_3_	SD
Age	130	31.18	23.00	40.00	28.00	32.00	33.00	4.031
Body height (cm)	130	166.45	150.00	183.00	163.00	167.00	170.00	5.940
Body weight (kg)	130	65.83	43.00	107.00	56.00	63.00	75.00	13.740
Gestational weight gain (GWG)	130	13.98	0.00	30.00	10.00	13.00	18.00	5.631
Week of the childbirth	130	39.26	33.00	43.00	38.00	39.00	41.00	1.914
Body birth length (cm)	130	54.98	45.00	62.00	54.00	56.00	57.00	3.155
Body birth weight (g)	130	3353.17	1800.00	4350.00	3051.00	3300.00	3700.00	492.967
Pain after childbirth	130	6.15	1.0	10.0	4.0	7.0	8.0	2.73
Apgar point	130	9.61	7.0	10.0	9.0	10.0	10.0	0.7

Max.—maximum value; Me—median; Min.—minimum value; N—number of participants; Q_1_—lower quartile; Q_3_—upper quartile; SD—standard deviation; x—Average.

**Table 2:** Level of prenatal and postnatal depression in the study group.

Depression EPDS	Before Childbirth		After Childbirth	
	N	%	N	%
0–10	108	83.08	78	60.0
>11	22	16.92	52	40.0
Total	130	100	130	100
Significance (*p*)	*p* = 0.009			

**Table 3:** Level of prenatal and postnatal anxiety in the study group.

Level of Generalized Anxiety (GAD7)	Before Childbirth		After Childbirth	
	N	%	N	%
Mild (0–4 pts)	56	43.08	50	38.46
Moderate (5–9 pts)	26	20.0	26	20.0
Moderately severe (10–14 pts)	20	15.38	16	12.31
Severe (15–21 pts)	28	21.54	38	29.23
Total	130	100	130	100
Significance (*p*)	*p* = 0.330			

**Table 4:** The influence of selected factors on a change in the level of prenatal and postnatal anxiety and depression in mothers.

Variables	N (%)	GAD 7			EPDS		
		X Before	X After	*p*	X Before	X After	*p*
Educational status							
Secondary	2 (1.5)	8.67	7.92	0.682	7.75	10.25	0.083
Professional	24 (18.5)	16.0	17.0	-	8.0	17.0	-
Higher	104 (80.0)	7.48	8.83	0.216	5.94	11.04	<0.001
Type of work							
Mental	80 (61.5)	7.68	8.13	0.667	5.88	10.38	<0.001
Physical	22 (16.9)	8.45	10.73	0.405	5.91	12.0	0.021
Does not work	28 (21.5)	7.79	9.14	0.582	7.86	11.93	0.072
Place of residence							
Urban	98 (75.4)	7.69	8.90	0.271	6.71	11.57	<0.001
Rural	32 (24.6)	8.25	8.44	0.917	5.06	9.19	0.011
SES							
Very good	38 (29.2)	6.37	8.11	0.165	6.32	10.32	0.001
Good	76 (58.5)	7.74	8.08	0.810	6.11	10.29	0.001
Average	16 (12.3)	11.75	13.75	0.306	7.25	15.88	0.004
Anxiety disorders in the examined person							
Yes	26 (20.0)	11.0	11.0	1.0	9.69	15.0	0.020
No	104 (80.0)	7.04	8.23	0.199	5.46	9.98	<0.001
Neurosis in the examined person							
Yes	28 (21.5)	9.86	8.79	0.649	8.93	13.07	0.012
No	26 (78.5)	7.27	8.78	0.132	5.59	10.41	<0.001
Depression in the examined person							
Yes	36 (27.7)	8.50	8.67	0.942	8.94	12.89	0.019
No	94 (72.3)	7.57	8.83	0.192	5.3	10.26	<0.001
Bipolar disorder in the examined person							
Yes	4 (3.1)	11.5	15.0	0.395	11.0	13.0	0.626
No	126 (96.9)	7.71	8.59	0.359	6.16	10.92	<0.001
Childbirth during pandemic							
Yes	54 (41.5)	8.59	8.26	0.835	6.26	11.07	<0.001
No	76 (58.5)	7.29	9.16	0.095	6.34	10.92	0.002
Visit after childbirth							
Yes	70 (53.8)	6.91	8.63	0.135	6.0	10.86	0.002
No	60 (46.2)	8.90	8.97	0.965	6.67	11.13	<0.001
Type of childbirth							
Natural	54 (41.5)	8.63	7.81	0.578	6.15	10.22	0.002
Cesarean section	76 (58.5)	7.26	9.47	0.065	6.42	11.53	<0.001
Assistance of medical personnel							
Yes	90 (69.2)	7.24	7.20	0.969	6.22	9.62	0.001
No	16 (12.3)	7.38	10.25	0.173	4.25	13.25	<0.001
Reluctantly	24 (18.5)	10.33	13.75	0.149	8.0	14.58	0.003
Pain making it difficult to care for the baby after birth							
Yes	60 (46.1)	9.63	12.43	0.037	7.37	13.33	<0.001
No	70 (53.9)	6.29	5.66	0.623	5.40	8.97	0.003
Problems with lactation							
Yes	66 (50.8)	6.94	10.88	0.004	6.06	13.15	<0.001
No	64 (49.2)	8.75	6.63	0.070	6.56	8.75	0.019

X after—average value in GAD7 or EPDS after childbirth; X before—average value in GAD7 or EPDS before childbirth.

## Data Availability

The data presented in this study are available on request from the corresponding author.

## Supplementary Materials

The following are available online at https://www.mdpi.com/article/10.3390/jcm10143193/s1, Figure S1: Responses to the statements of EPDS. Figure S2: Responses to the statements of GAD7.
