# Supplementary material for: Prenatal and Postnatal Anxiety and Depression in Mothers during the COVID-19 Pandemic
Source: J Clin Med. 2021 Jul 20;10(14):3193. doi: 10.3390/jcm10143193 (PMC8304229; doi:10.3390/jcm10143193)
Supplement: Supplementary file 1 [file jcm-10-03193-s001.zip › jcm-1279221-supplementary.pdf]

Figure S1: Responses to the statements of EPDS.

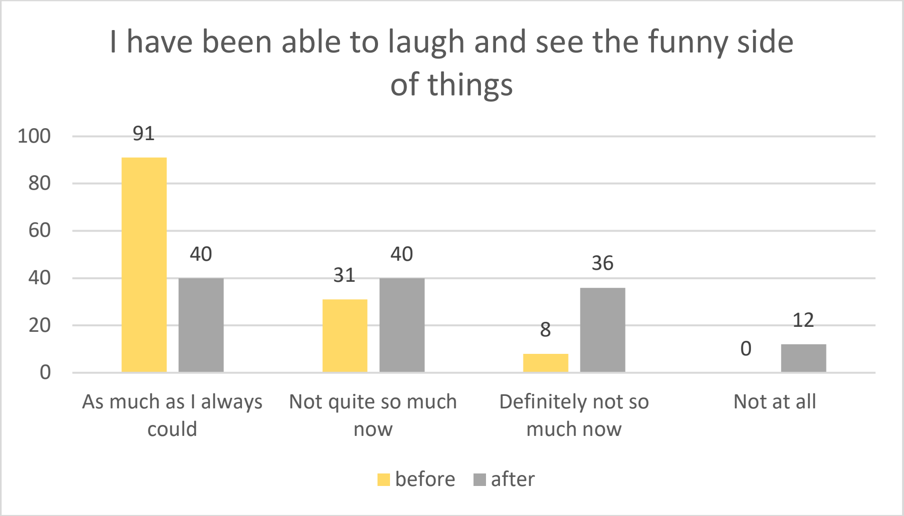

Figure S1a. I have been able to laugh and see the funny side of things.

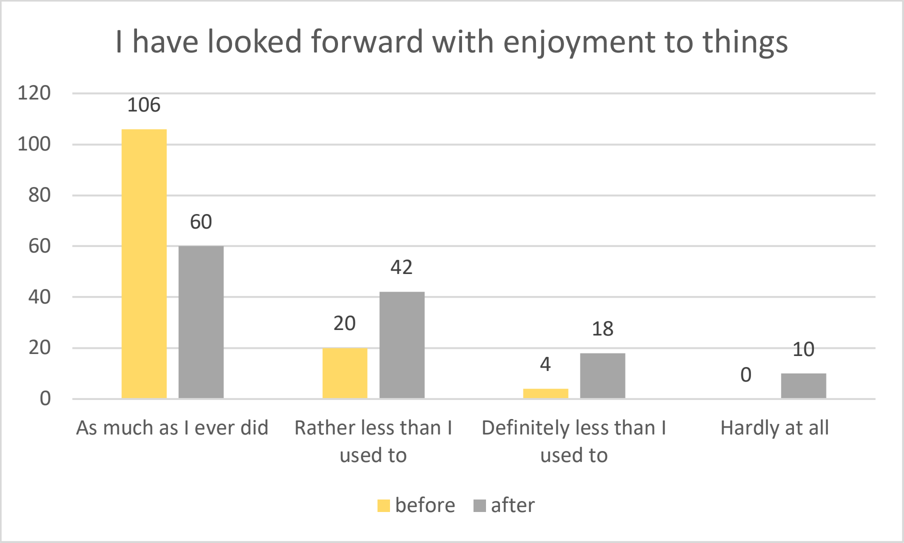

Figure S1b. I have looked forward with enjoyment to things.

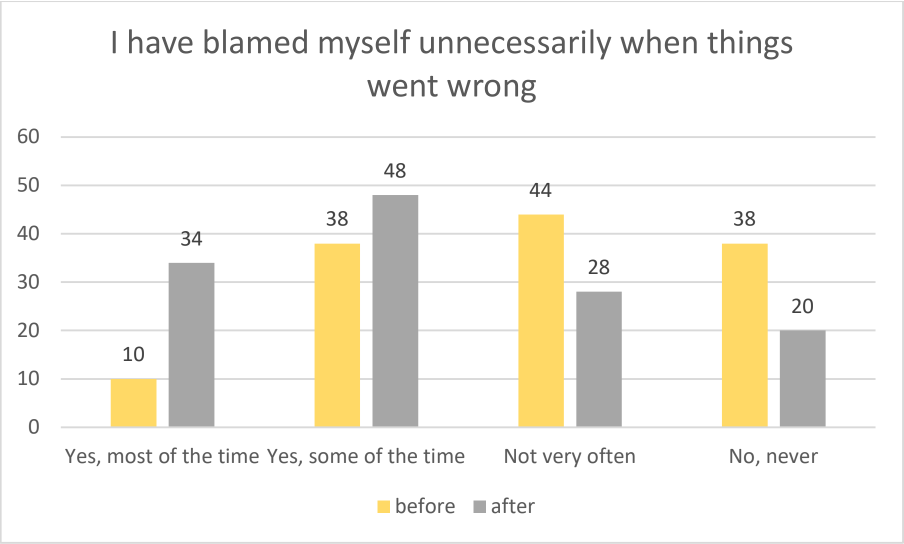

Figure S1c. I have blamed myself unnecessarily when things went wrong.

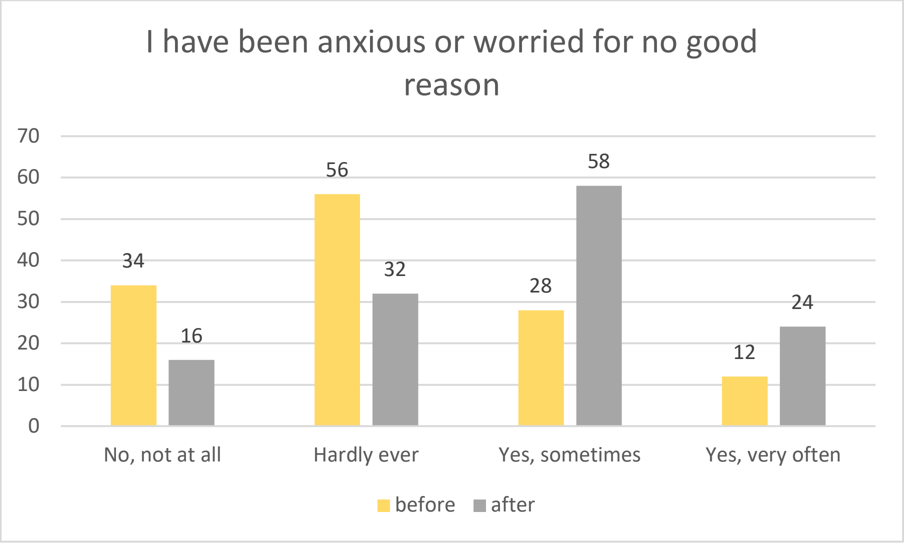

Figure S1d. I have been anxious or worried for no good reason.

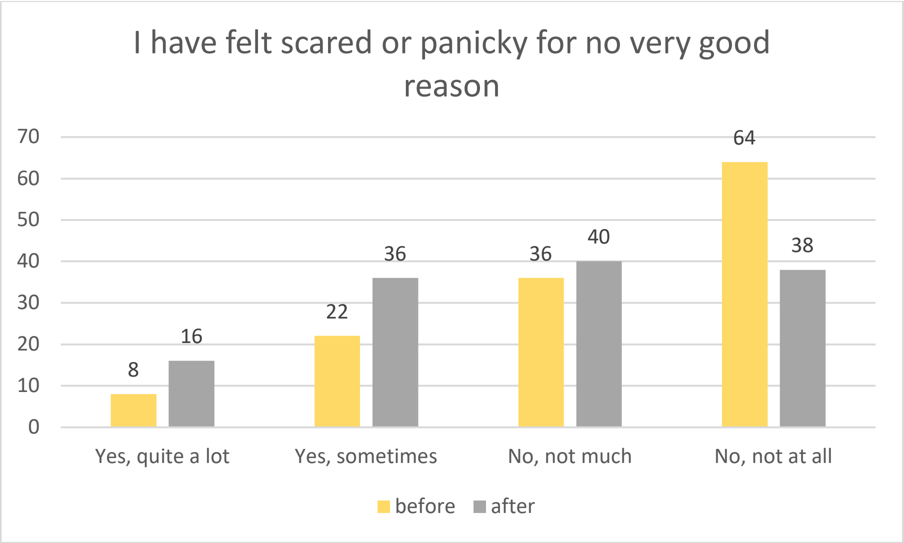

Figure S1e. I have felt scared or panicky without a good reason.

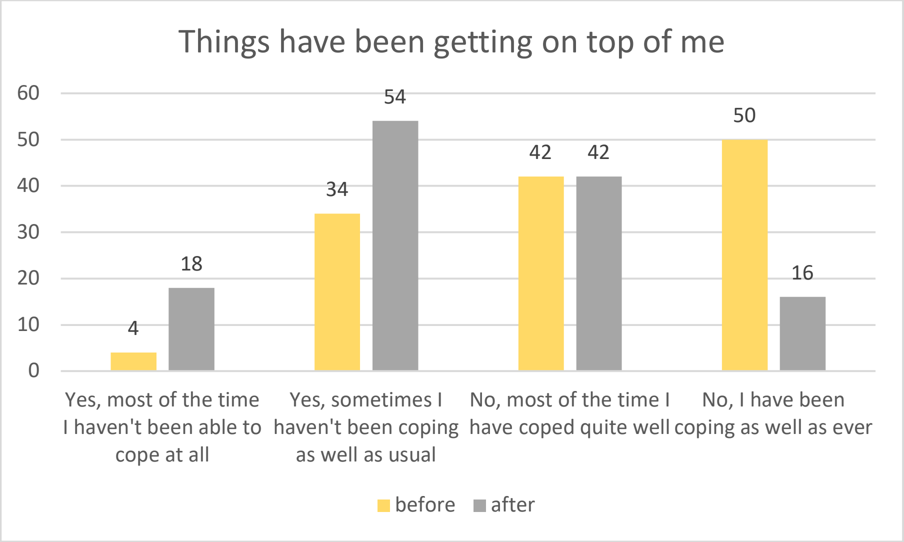

Figure S1f. Things have been getting on top of me.

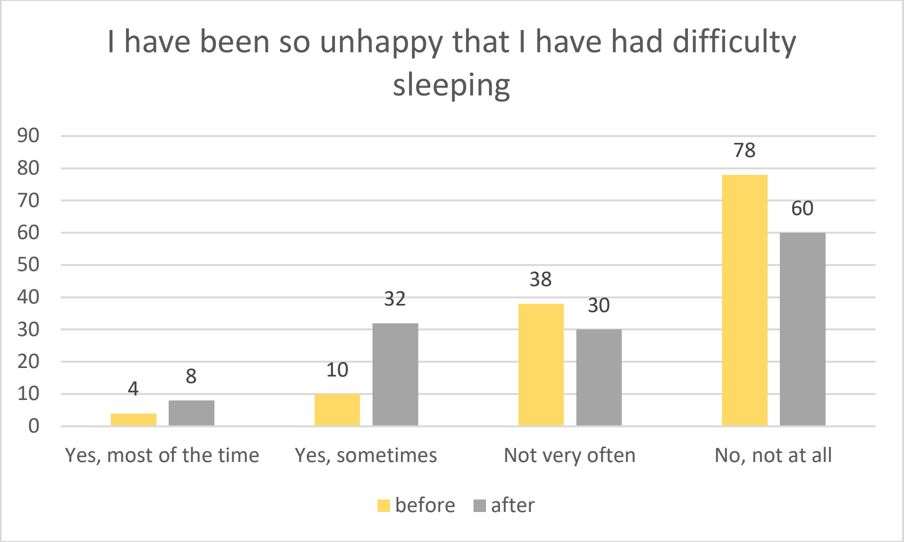

Figure S1g. I have been so unhappy that I have had difficulty sleeping.

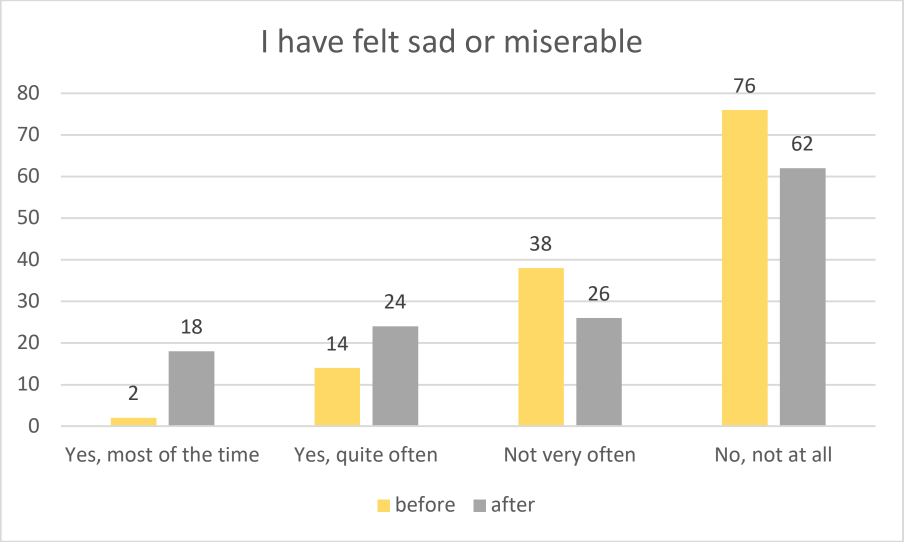

Figure S1h. I have felt sad or miserable.

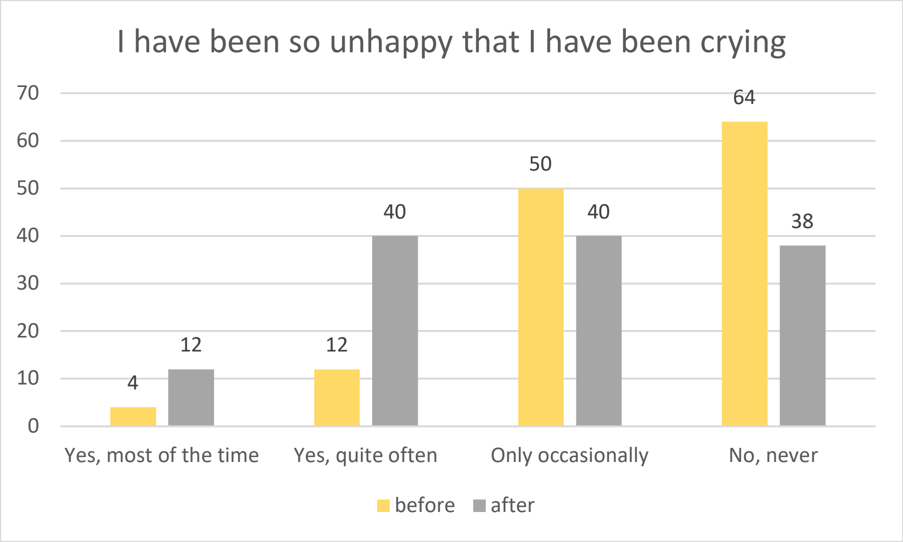

Figure S1i. I have been so unhappy that I have been crying.

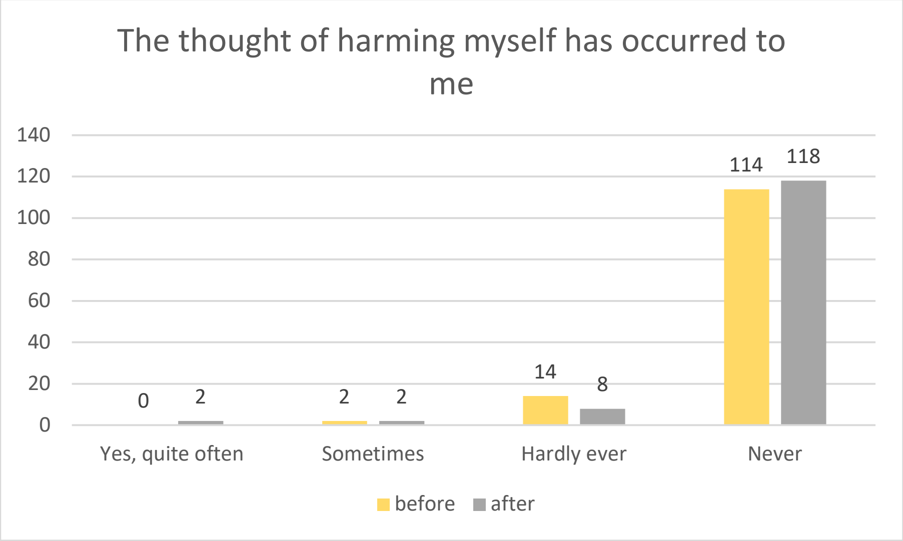

Figure S1j. The thought of harming myself has occurred to me.

Figure S2: Responses to the statements of GAD7.

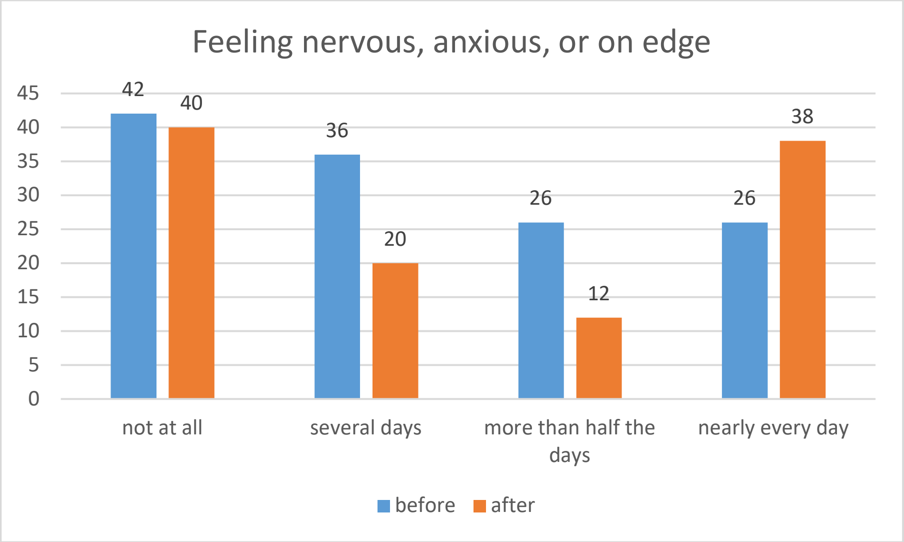

Figure S2a. Feeling nervous, anxious, or on edge.

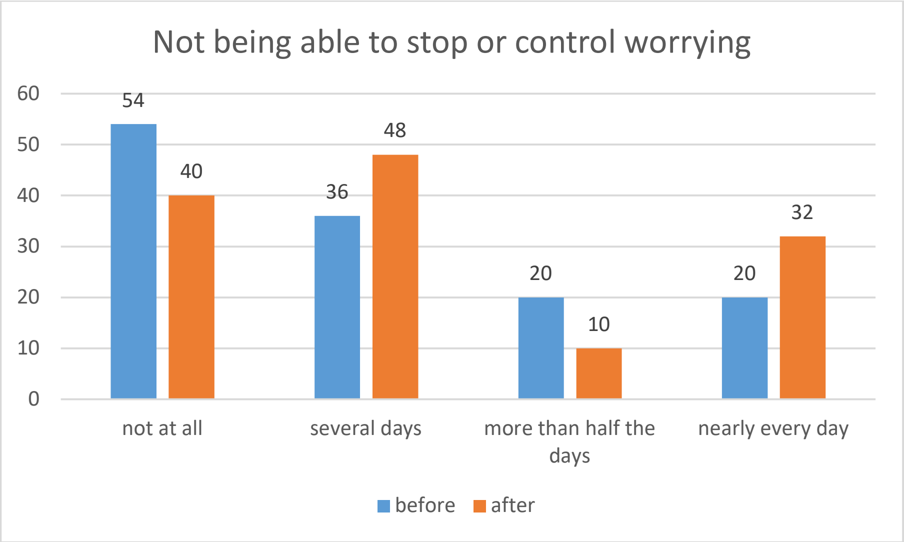

Figure S2b. Not being able to stop or control worrying.

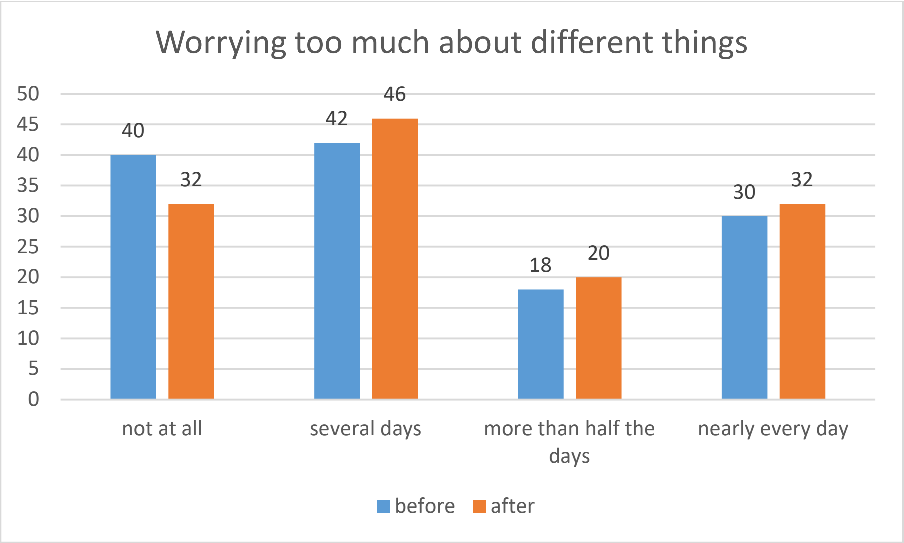

Figure S2c. Worrying too much about different things.

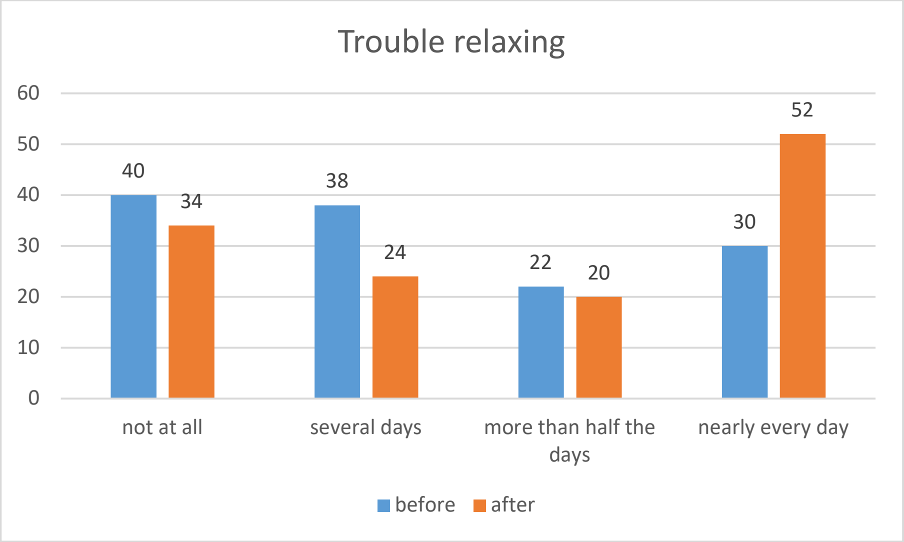

Figure S2d. Trouble relaxing.

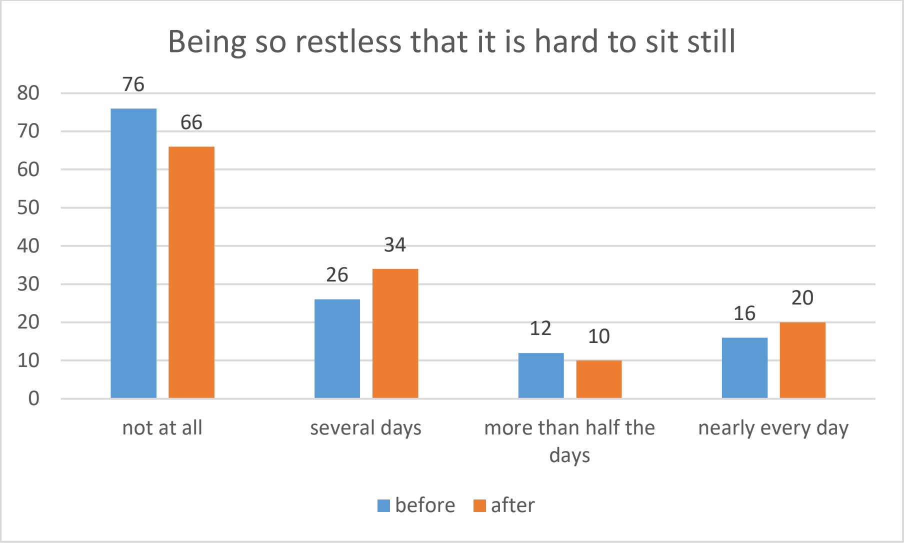

Figure S2e. Being so restless that it is hard to sit still.

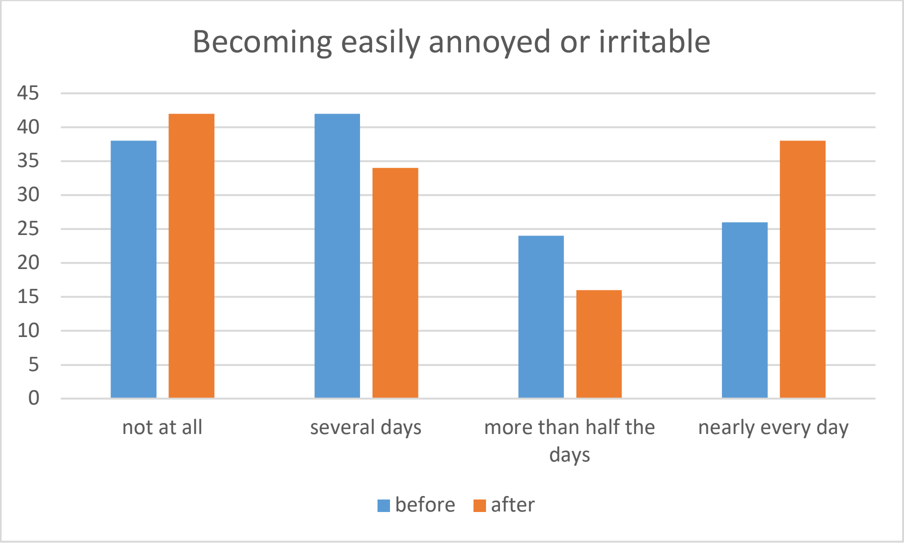

Figure S2f. Becoming easily annoyed or irritable.

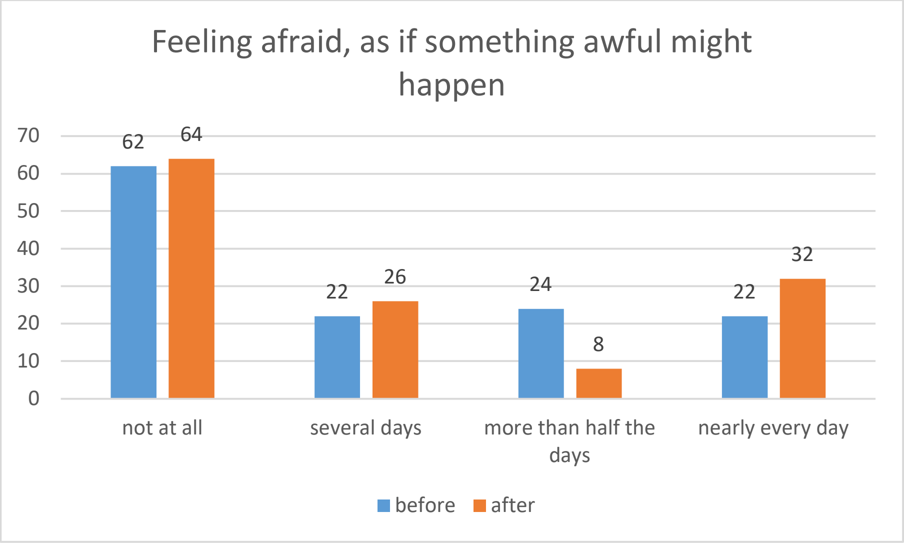

Figure S2g. Feeling afraid, as if something awful might happen.
